# Clozapine prevented social interaction deficits and reduced c-Fos immunoreactivity expression in several brain areas of rats exposed to acute restraint stress

**DOI:** 10.1371/journal.pone.0262728

**Published:** 2022-03-03

**Authors:** Rodolpho Pereira de Oliveira, José Simões de Andrade, Marianna Spina, João Vítor Chamon, Paulo Henrique Dias Silva, Ana Keyla Werder, Daniela Ortolani, Lucas de Santana Cardoso Thomaz, Simone Romariz, Daniel Araki Ribeiro, Beatriz Monteiro Longo, Regina Célia Spadari, Milena de Barros Viana, Liana Melo-Thomas, Isabel Cristina Céspedes, Regina Cláudia Barbosa da Silva

**Affiliations:** 1 Departamento de Biociências, Universidade Federal de São Paulo (UNIFESP), Santos (SP), Brazil; 2 Departamento de Fisiologia, Universidade Federal de São Paulo (UNIFESP/SP), São Paulo, Brazil; 3 Behavioral Neuroscience, Experimental and Biological Psychology, Philipps-Universityof Marburg, Marburg, Germany; 4 Marburg Center for Mind, Brain, and Behavior (MCMBB), Marburg, Marburg, Germany; 5 Departamento de Morfologia e Genética, Universidade Federal de São Paulo (UNIFESP), São Paulo, Brazil; 6 Instituto de Neurociências e Comportamento (INeC), Ribeirão Preto, São Paulo, Brazil; Mayo Clinic, UNITED STATES

## Abstract

In the present study, we evaluate the effect of acute restraint stress (15 min) of male Wistar rats on social interaction measurements and c-Fos immunoreactivity (c-Fos-ir) expression, a marker of neuronal activity, in areas involved with the modulation of acute physical restraint in rats, i.e., the paraventricular nucleus of the hypothalamus (PVN), median raphe nucleus (MnR), medial prefrontal cortex (mPFC), cingulate prefrontal cortex (cPFC), nucleus accumbens (NaC), hippocampus (CA3), lateral septum (LS) and medial amygdala (MeA). We considered the hypothesis that restraint stress exposure could promote social withdrawal induced by the activation of the hypothalamic-pituitary-adrenocortical (HPA) axis, and increase c-Fos expression in these limbic forebrain areas investigated. In addition, we investigated whether pretreatment with the atypical antipsychotic clozapine (5 mg/kg; I.P.) could attenuate or block the effects of restraint on these responses. We found that restraint stress induced social withdrawal, and increased c-Fos-ir in these areas, demonstrating that a single 15 min session of physical restraint of rats effectively activated the HPA axis, representing an effective tool for the investigation of neuronal activity in brain regions sensitive to stress. Conversely, pretreatment with clozapine, prevented social withdrawal and reduced c-Fos expression. We suggest that treatment with clozapine exerted a preventive effect in the social interaction deficit, at least in part, by blocking the effect of restraint stress in brain regions that are known to regulate the HPA-axis, including the cerebral cortex, hippocampus, hypothalamus, septum and amygdala. Further experiments will be done to confirm this hypothesis.

## 1. Introduction

Stress is a natural biological response that enables individuals to cope with environmental stressors. The stress response activates the autonomic nervous system and the hypothalamic-pituitary-adrenocortical (HPA) axis [1]. The deriving increase in catecholamines and glucocorticoids triggers proper behavioral and physiological reactions [1, 2] that depend on the type, intensity and time of exposure to the stressor stimulus [3]. Although being a reaction aimed to guarantee survival, exposition to stressful events is associated to an increased risk to develop stress-related psychopathology [4, 5].

Exposure of rodents to different stress paradigms induces behavioral and physiological changes [6–8]. For example, physical restraint is considered a very effective sort of inescapable stress [9]. It results from the aversive nature of remaining immobile and induces increases in adrenocorticotropic hormone and corticosterone [9].

The social interaction (SI) test reveals the natural trend of rodents to maintain social contact with their conspecifics [10]. Thus, lack of interaction is regarded as social withdrawal, a common feature of stress related disorders as for instance schizophrenia [10].

Some studies have investigated the effect of atypical antipsychotics in response to acute stress in rats. For instance, Marchisella et al. [11] evaluated the effects of treatment with the antipsychotic blonanserin on the rat’s ability to respond to a single 5 min session of forced swim stress (FSS). They investigated the expression levels of inducible early genes (IEGs) including c-Fos in regions such as the prefrontal cortex, striatum, hippocampus, and hypothalamus. The results demonstrated that FSS produced a significant elevation of IEGs transcription in different brain regions while the response of blonanserin-treated rats to FSS showed that the upregulation of IEGs was greatly reduced in the striatum, but was preserved in the prefrontal cortex and the ventral hippocampus. They concluded that the differential activation of specific brain regions under challenging conditions may contribute to specific clinical features of the drug. Fumagalli et al. [12] used the same FSS protocol to investigate the ability of the antipsychotic lurasidone to modulate the neurotrophin BDNF expression in hippocampus and prefrontal cortex of rats. They found that the modulation of BDNF mRNA levels in response to acute FSS in lurasidone-treated rats was significantly enhanced in the hippocampus, and to less extent in the prefrontal cortex, through the selective regulation of different neurotrophin isoforms, suggesting that the adaptative changes induced by the treatment with lurasidone may contribute to the improvement of cognitive functions.

The aim of this study was to assess the effect of acute restraint stress (15 min) in social behavior, and c-Fos immunoreactivity in areas associated with the stress response, such as hypothalamus, median raphe nucleus, prefrontal cortex, nucleus accumbens, hippocampus, septum and amygdala. We considered the hypothesis that restraint stress exposure could promote social withdrawal induced by the activation of the HPA axis, and increase c-Fos expression in these limbic forebrain areas investigated. In addition, aiming to investigate whether the same pathway is activated both in psychosis and stress we tested the ability of clozapine, a serotonin 2A/2C antagonist, to prevent possible deficits in the social interaction and attenuate or block c-Fos expression in these limbic forebrain areas of rats submitted to the acute restraint stress. Clozapine was chosen because clinically it is one of the most commonly used antipsychotic and due to its superior efficacy among all antipsychotic agents [13].

## 2. Materials and methods

### 2.1. Animals

A total of 60 naïve male Wistar rats provided by CEDEME—Federal University of São Paulo, weighing 300–350 g (10 weeks old) at the beginning of the experiments, were used for all experiments. As reported previously [14] following arrival, the animals remained undisturbed for one week, for acclimatization. They were housed in groups of four in standard plastic cages (45 cm x 35 cm x 15 cm) with wood chip bedding, under a 12:12 dark/light cycle (lights on at 07:00 am) under standard conditions in a temperature (22 ± 1°C) and humidity (55 ± 5%) controlled room and with food and water given *ad libitum* throughout the extent of the study. The experiments were conducted during the light phase of the light/dark cycle, between 12:00 and 17:00 h. All experiments were performed in compliance with the recommendations of the SBNeC (Brazilian Society for Neuroscience and Behavior), which follows the National Institutes of Health (NIH) guide for the care and use of laboratory animals. This work has also been approved by the Ethics Committee on Animal Use from the Federal University of São Paulo (Protocol no. 8412060315).

### 2.2. Drug treatment

Clozapine (Novartis, Brazil) was obtained in a commercial form for intravenous use, and was dissolved with physiological saline solution (0.9% sodium chloride) to obtain the required concentration of 5 mg/ml. It was given intraperitoneally (I.P.) at a dose of 5 mg/kg in a volume of 1 ml/kg body weight. Controls received an equivalent volume of physiological saline [14]. Experiments were conducted 45 min after clozapine I.P. injection when its peak level was reached in the rat brain [15].

### 2.3. Experimental groups

Independent groups of rats were used for social interaction (SI) test and immunohistochemical analysis. The reason for that was to ensure that we were indeed only observing the effect of the restraint stress on social interaction behavior and on c-Fos expression, respectively, avoiding a possible interference of the social interaction test itself on c-Fos expression.

We considered stressed group the one in which the animals underwent the acute restraint procedure described in the subtopic 2.4.1. During the SI test, four groups of rats were used: saline/non-stressed (N = 10); saline/stressed (N = 10); clozapine/non-stressed (N = 10), and clozapine/stressed (N = 10). In order to investigate the immunoreactivity to the c-Fos protein in areas activated by the acute restraint procedure, four independent groups of rats were used saline/non-stressed (N = 5); saline/stressed (N = 5); clozapine/non-stressed (N = 5) and clozapine/stressed (N = 5).

### 2.4. Procedures

#### 2.4.1. Acute restraint

Rats were restrained for 15 min by the use of an acrylic restraining cylinder (63 ×65 ×232 mm; Insight Equipment, Ribeirão Preto, SP, Brazil) adjustable to the size of the animal and with holes on the sides to provide ventilation [9]. After this period, they were individually reassigned to their home cage for further 15 min before the behavioral tests were performed [9]. This period was due since pilot studies showed that the animals engaged in auto-grooming behavior immediately after leaving the restraining cylinder [16]. The restraining procedure took place in the same experimental room where the behavioral tests were subsequently conducted. Control animals were left undisturbed in their home cages, in the same experimental room and for the same amount of time before behavioral measurements [9].

#### 2.4.2. Social interaction (SI) test

**Fig 1:**
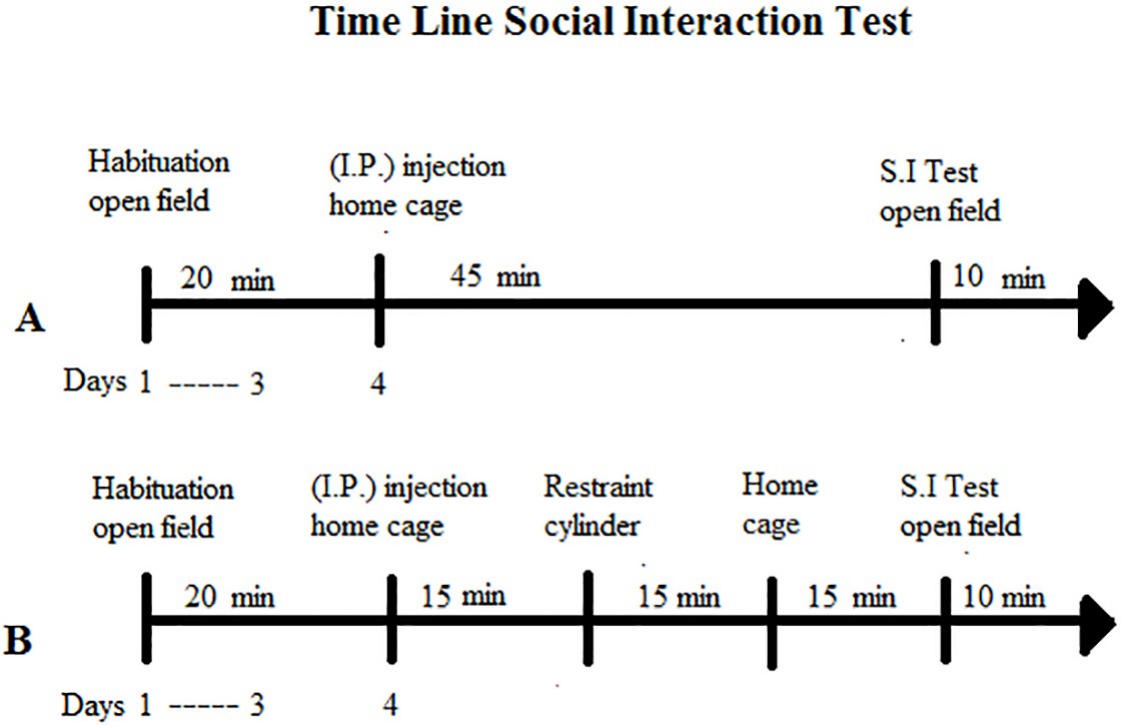
Schematic timeline illustration. The rats from the non-stressed group (A) (saline or clozapine) and stressed group (B) (saline or clozapine) were habituated individually to the open field for 20 min for three consecutive days. On the fourth day, they received I.P. injection of saline or clozapine according to the drug-treatment group they belonged. In sequence, the non-stressed group of rats remained in their home cage for 45 min and then underwent the SI test in the open field for 10 min. The stressed group of rats remained in their home cage for 15 min. After this, they were restrained for 15 min into the acrylic restraining cylinder and then placed back in their home cage for the same amount of time. They were tested for SI test 45 min after clozapine injection.

The tests were carried out in an open field, which consists of a circular arena made of Plexiglas (60 cm in diameter and50 cm height, the surface of the floor being divided in 12 equal sections) situated in an experimental room (60 lx at the floor level of the arena) [17]. The rats were habituated to the open field for 20 min for three consecutive days before the behavioral test. On the fourth day, the rats from the stressed groups (saline/stressed and clozapine/stressed) received I.P. injection of saline or clozapine, and were placed in their home cage for 15 min. In sequence, they were restrained for 15 min into the acrylic restraining cylinder and then placed back in their home cage for the same amount of time. Thus, 15 min after the acute restraint procedure, and 45 min after clozapine injection, when the drug peak level was reached in the rat brain [15] they were exposed to the SI test (Fig 1A and 1B. Schematic timeline illustration). The rats were matched on the basis of body weights (animal weight did not differ by more than fifteen grams) and placed simultaneously in pairs in the open field for 10 min. The component of the pair was another unfamiliar co-specific naïve rat (stimulus rat which did not receive any drug-treatment). Rat behavior was recorded and analyzed later using Ethovision software (Noldus Information Technology, Netherlands). The experimenter then scored the amount of time that the stressed-rats spent in social interaction described as time spent sniffing (investigative sniffing the snout, body, or anogenital region of the stimulus rat), following (moves behind the stimulus rat), climbing (climbing over the back or pushing the head and/or forepart beneath of the stimulus rat) [18]. The medium interaction time of each experimental group was used for statistical analysis [18]. Animals of the non-stressed groups (saline/non-stressed and clozapine/non stressed) did not undergo the acute restraint procedure. Thus, 45 min after receiving I.P. injection of saline or clozapine, they underwent the SI test as described before. Locomotor activity was scored based on the number of crossings (section crossed with the four paws) the animals performed in the open field [17]. After each experimental session, the open field was cleaned with a 10% ethanol solution [9].

#### 2.4.3. Immunohistochemical analysis

c-Fos was adopted to show the regions associated with the stress response induced by the acute restraint. Rats belonging to saline or clozapine stressed groups received I.P. injections of saline or clozapine, respectively, and 15 min later underwent the acrylic restraining cylinder (same procedure described in the item 2.4.1.) [9]. After thirty min, from the beginning of the restraint procedure, they were euthanized. The control rats in the non-stressed groups received an I.P. injection of saline or clozapine, respectively, and were left undisturbed in their home cages at the same experimental room where the behavioral tests were conducted, and for the same period of time as the stressed groups, before they were euthanized [9].

The animals were weighed and anesthetized with ketamine/xylazine 2:1 (1 ml/kg) and perfused with ≈100 ml of 0.9% saline for approximately 1 min, followed by 500–700 ml of 4% formaldehyde (at 4°C), pH 9.5, for approximately 25 min. Their brains were post-fixed for 1 h in the same fixative solution, and then stored in a solution containing 20% sucrose for cryoprotection at 4°C. Regularly spaced series (5 × 1-in-5) of 30 μm-thick frozen sections were cut in the frontal plane, collected in ethylene glycol-based cryoprotectant solution and stored at -20°C for later determination of c-Fos-ir [9].

A series of brain slices was pretreated with hydrogen peroxide (3%; Sigma, St. Louis, MO, USA) before addition of the primary antibody to squelch endogenous peroxidase activity in the tissue. c-Fos neurons were identified by using a polyclonal anti-serum raised in rabbit (anti-c-Fos, 1:20,000; Oncogene, Cambridge, MA, USA equivalent to ABE457 Merck Millipore, Darmstadt, Germany) as primary antibody. In sequence, the slices were incubated with biotinylated anti-rabbit secondary antibody (goat; 1:1000). Immunohistochemical was performed using a conventional avidin-biotin immunoperoxidase protocol [19] and Vectastain Elite reagents (Vector Laboratories^®^, Burlingame, CA, USA). The reaction with diaminobenzidine-DAB chromogen (0.05%; Sigma^®^) was amplified using nickel ammonium sulfate, producing a black color labeling [9, 20, 21].

The sections were then mounted on gelatin-coated slides, allowed to dry for approximately 18 h and counterstained with thionin in order to visualize the labeled cells within the borders of each nucleus. Adjoining series of sections were stained with 0.25% thionin for cytoarchitectural reference purposes [9]. c-Fos cells were quantified in sections, having as reference the following AP coordinates [22]: Bregmas: paraventricular nucleus of the hypothalamus (PVN) (-1,92 mm), median raphe nucleus (MnR) (-7.56 mm), medial prefrontal cortex (mPFC) (+1.68 mm), cingulate prefrontal cortex (cPFC) (+1.68 mm), nucleus accumbens (NaC) (+0.70 mm), hippocampus (CA3) (-2.28 mm), lateral septum (LS) (+0.96 mm), and medial amygdala (MeA)(-2.76 mm). Cells were considered immunoreactive to c-Fos when their nuclei were strongly stained. The dark stained cells were counted in five fields captured for each region for each group, according to the bregma orientation of the Paxinos Atlas. For the semi-quantitative analysis, sections were analyzed under bright-field illumination using the Image-Pro Plus ^™^ software (Media Cybernetics^®^, Silver Spring, MD, USA), with a Zeiss—Axio Observer D1 microscope by an observer blind to the treatment conditions [9]. The color spectrum utilized in the measurements was adjusted from dark brown to black, and a fixed bi-dimensional area (500 μm x 500 μm) was adopted for each region of interest. Therefore, the graphs represent the number of c-Fos cells per field. To determine whether the primary or the secondary antibodies produced false-positive results, controls with omission of primary and secondary antibodies were performed [23]. No specific c-Fos labelling was detected in brain regions of stressed rats evaluated in this study.

### 2.5. Statistical analysis

All sample data were submitted to the Shapiro-Wilk normality test. Behavioral data from the social interaction test (experiment 1), and number of c-Fos cells in each neural structure (experiment 2) were submitted to a two-way analysis of variance (ANOVA), followed by the Bonferroni’s *post hoc* test (software R Core Team, 2020). The condition (non-stressed and stressed) and the treatment (saline or clozapine) were considered as factors. For all tests, values of P ≤ 0.05 were considered significant.

## 3. Results

### 3.1. Behavioral measurements

#### 3.1.1. Social interaction and locomotor activity

**Fig 2:**
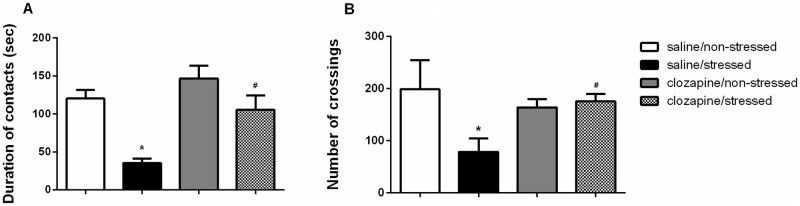
Data are presented as mean ± SEM. A) Duration of contacts and B) number of crossings for non-stressed groups of rats (saline and clozapine) and for stressed groups of rats submitted to the physical restraint procedure and pretreated with saline or clozapine. N = 10 for each one of the following groups: saline/non-stressed; saline/stressed; clozapine/non-stressed and clozapine/stressed. Group saline/stressed as compared to saline/non-stressed *P<0.001; Group clozapine/stressed as compared to saline/stressed ^#^P<0.001. Two-way ANOVA, followed by Bonferroni’s post hoc test.

Fig 2A and 2B show total duration of contacts and number of crossings, respectively, for non-stressed and stressed rats pretreated with saline or clozapine.

Regarding the total duration of contacts, two-way ANOVA revealed a significant effect of the condition factor in the time spent in contacts [F(1, 36) = 30.48; P<0.001]. *Post hoc* test showed that the saline/stressed group presented a decrease in the total duration of contacts in comparison to the saline/non-stressed group (P = 0.001) demonstrating a significant effect of acute restraint. There was no difference between the clozapine/non-stressed and the clozapine/stressed groups (P = 0.318). In the treatment factor, it was observed a significant main effect [F (1,36) = 17.00; P<0.001]. *Post hoc* test showed that the clozapine/stressed group presented an increase in the total duration of contacts in comparison to the saline/stressed group (P<0.001) demonstrating that the pretreatment with clozapine prevented acute restraint-induced disruption of social interaction. There was no difference between the saline/non-stressed and the clozapine/non-stressed groups (P = 0.875). It was observed a significant main effect of the interaction between condition and treatment factors [F(1,36) = 9.35; P = 0.004].

Regarding the locomotor activity, two-way ANOVA revealed a significant effect of the condition factor in the number of crossings [F(1, 36) = 5.02; P<0.050]. *Post hoc* test showed that the saline/stressed group presented a decrease in the number of crossings in the open field compared to the saline/non-stressed group (P = 0.001). There was no difference between the clozapine/non-stressed and the clozapine/stressed groups (P = 0.999). In the treatment factor, it was observed a significant main effect [F (1,36) = 24.06; P<0.001]. *Post hoc* test showed that the clozapine/stressed group presented an increase in the number of crossings in comparison to the saline/stressed group (P = 0.001). There was no significant difference between the saline/non-stressed and clozapine/non-stressed groups (P = 0.194), thus discarding any motor effect of the clozapine treatment. It was observed a significant main effect of the interaction between condition and treatment factors [F(1,36) = 26.23; P = 0.001].

### 3.2. c-Fos protein immunoreactivity

The quantitative analyses of c-Fos-ir in the analyzed brain areas of non-stressed and stressed rats pretreated with saline or clozapine are described for each region in Figs 3–10.

**Fig 3:**
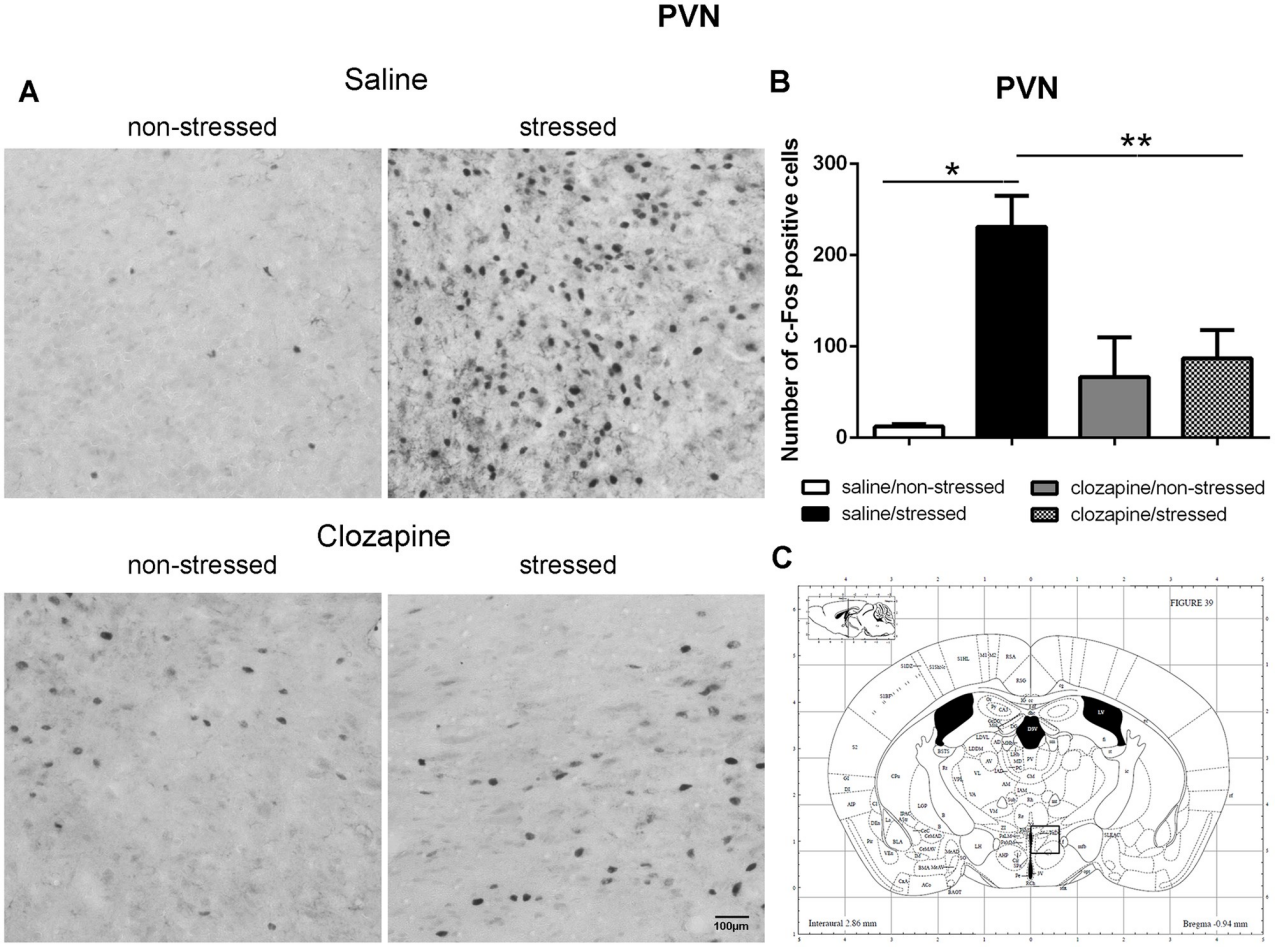
c-Fos immunoreactive cells photomicrographs and quantification in the paraventricular nucleus of the hypothalamus (PVN). (A) c-Fos staining (dark spots) in coronal sections of PVN of the four groups: saline/non-stressed, saline/stressed, clozapine/non-stressed, clozapine/stressed. Scale bar: 100 μm. (B) The c-Fos cell counting in the PVN revealed a higher number of c-Fos cells in saline/stressed group (two-way ANOVA, *P <0.05; **P <0.005), data showed as mean ± S.E.M. (C) Representative rat brain atlas drawing [22]. Localization of the brain area selected for the counting of c-Fos cells indicated in the square.

**Fig 4:**
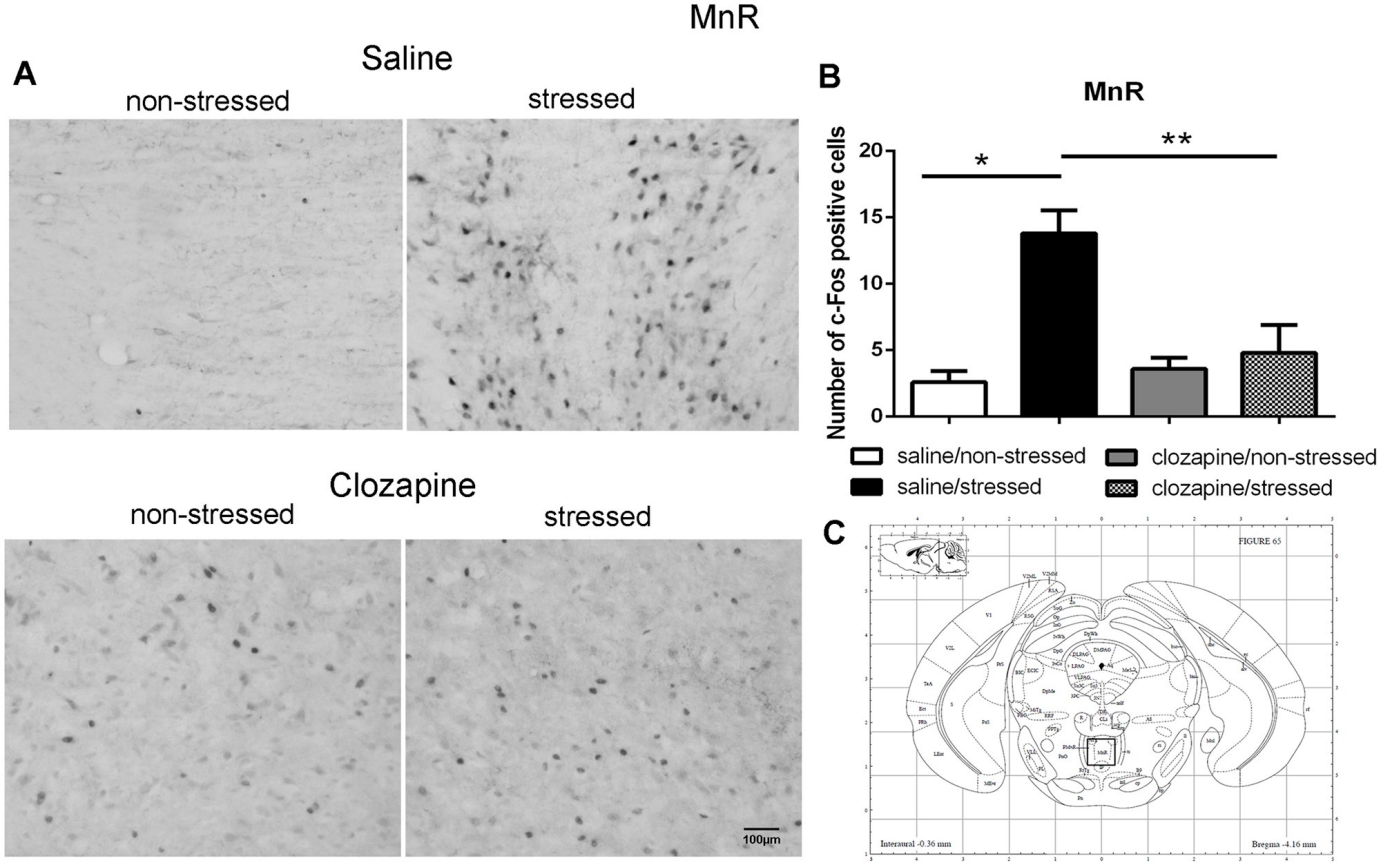
c-Fos immunoreactive cells photomicrographs and quantification in the median raphe nucleus (MnR). (A) c-Fos staining (dark spots) in coronal sections of MnR of the four groups: saline/non-stressed, saline/stressed, clozapine/non-stressed, clozapine/stressed. Scale bar: 100 μm. (B) The c-Fos cell counting in the MnR revealed a higher number of c-Fos cells in saline/stressed group (two-way ANOVA, *P <0.05; **P <0.005), data showed as mean ± S.E.M. (C) Representativeratbrain atlas drawing [22]. Localization of the brain area selected for the counting of c-Fos cells is indicated in the square.

**Fig 5:**
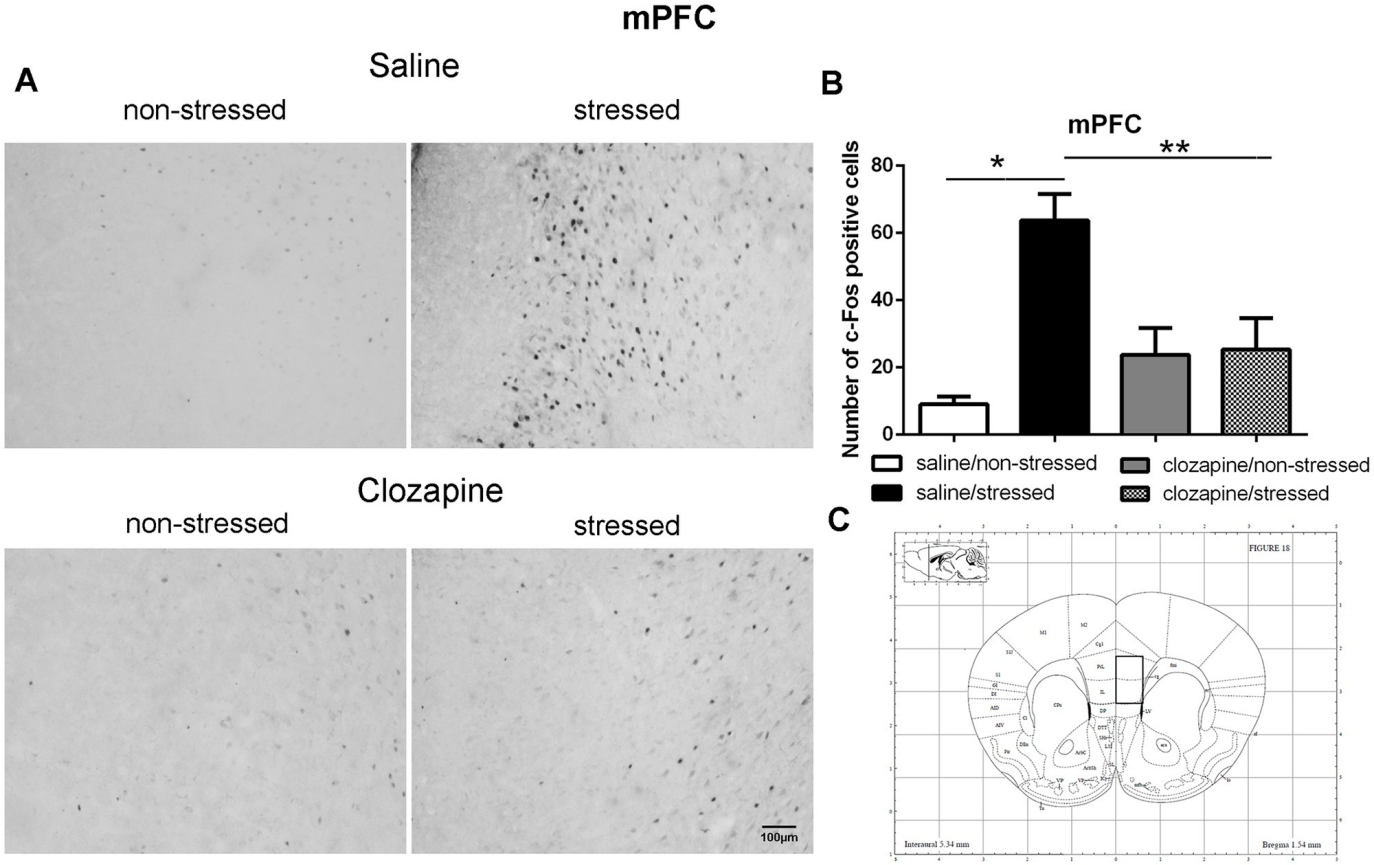
c-Fos immunoreactive cells photomicrographs and quantification in the medial prefrontal cortex (mPFC). (A) c-Fos staining (dark spots) in coronal sections of mPFC of the four groups: saline/non-stressed, saline/stressed, clozapine/non-stressed, clozapine/stressed. Scale bar: 100 μm. (B) The c-Fos cell counting in the mPFC revealed a higher number of c-Fos cells in saline/stressed group (two-way ANOVA, *P <0.05; **P <0.005), data showed as mean ± S.E.M. (C) Representative rat brain atlas drawing [22]. Localization of the brain area selected for the counting of c-Fos cells is indicated in the square.

**Fig 6:**
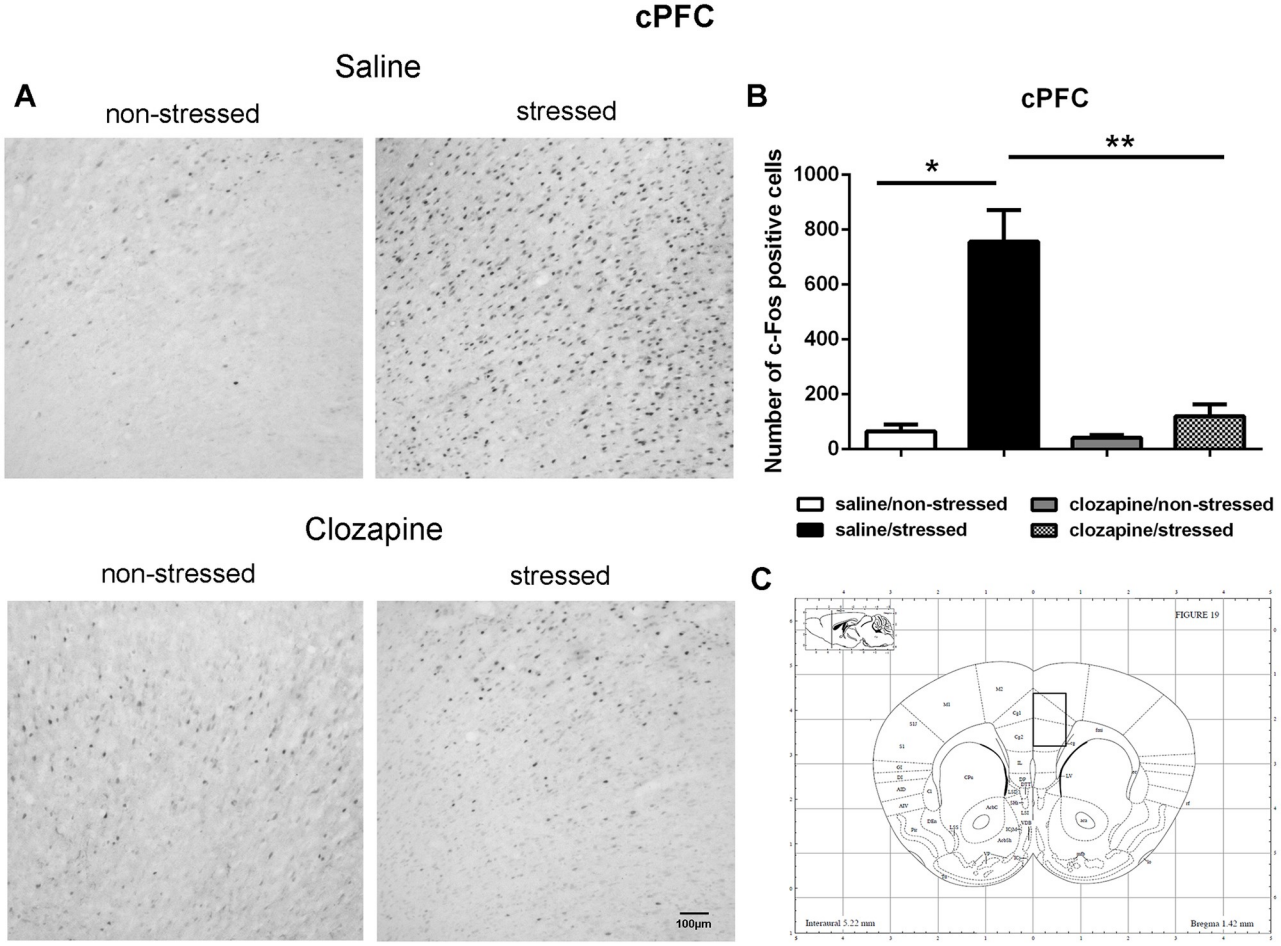
c-Fos immunoreactive cells photomicrographs and quantification in the cingulate prefrontal cortex (cPFC). (A) c-Fos staining (dark spots) in coronal sections of cPFC of the four groups: saline/non-stressed, saline/stressed, clozapine/non-stressed, clozapine/stressed. Scale bar: 100 μm. (B) The c-Fos cell counting in the cPFC revealed a higher number of c-Fos cells in saline/stressed group (two-way ANOVA, *P <0.05; **P <0.005), data showed as mean ± S.E.M. (C) Representative rat brain atlas drawing [22]. Localization of the brain area selected for the counting of c-Fos cells is indicated in the square.

**Fig 7:**
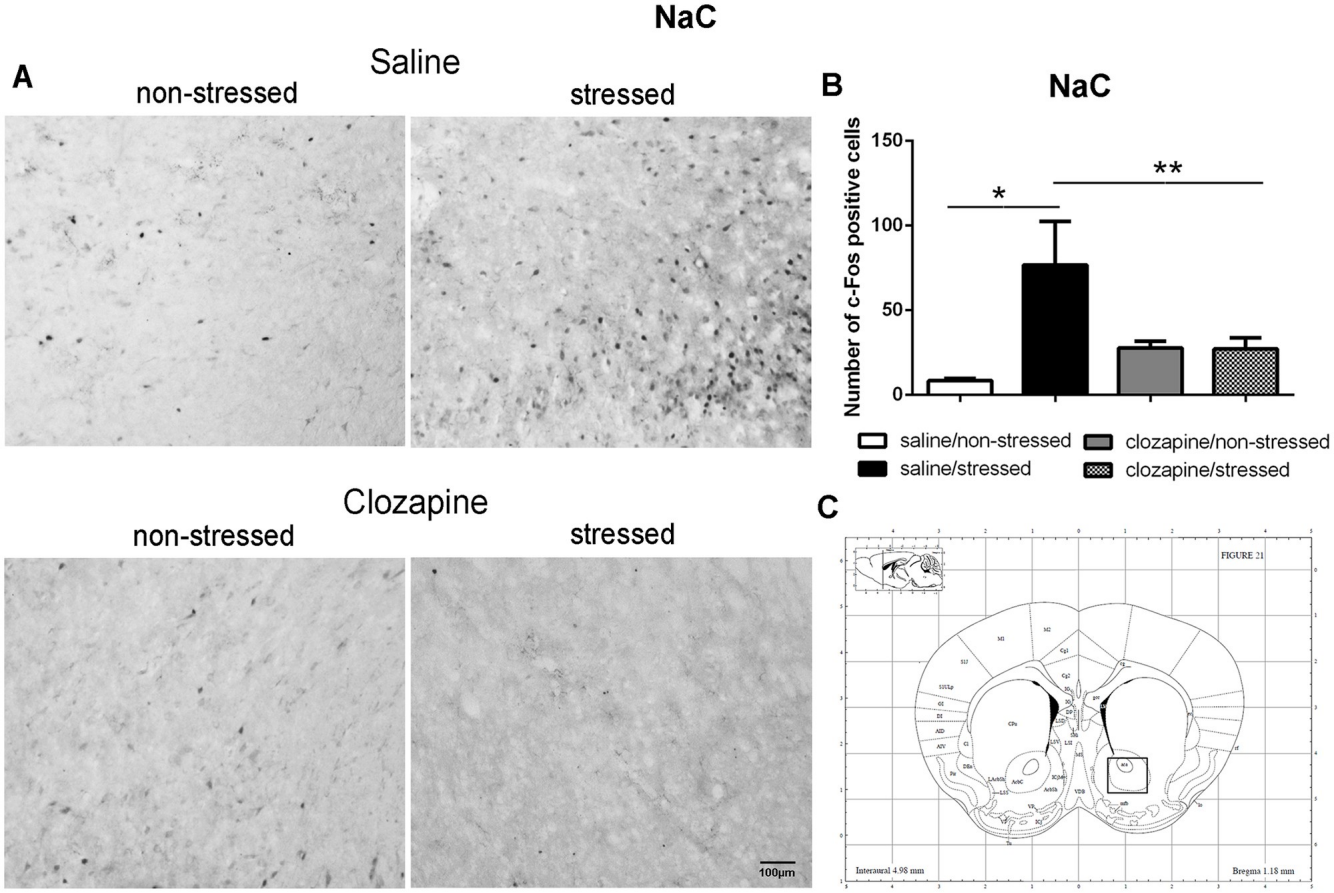
c-Fos immunoreactive cells photomicrographs and quantification in the nucleus accumbens (NaC). (A) c-Fos staining (dark spots) in coronal sections of NaC of the four groups: saline/non-stressed, saline/stressed, clozapine/non-stressed, clozapine/stressed. Scale bar: 100 μm. (B) The c-Fos cell counting in the NaC revealed a higher number of c-Fos cells in saline/stressed group (two-way ANOVA, *P <0.05; **P <0.005), data showed as mean ± S.E.M. (C) Representative rat brain atlas drawing [22]. Localization of the brain area selected for the counting of c-Fos cells is indicated in the square.

**Fig 8:**
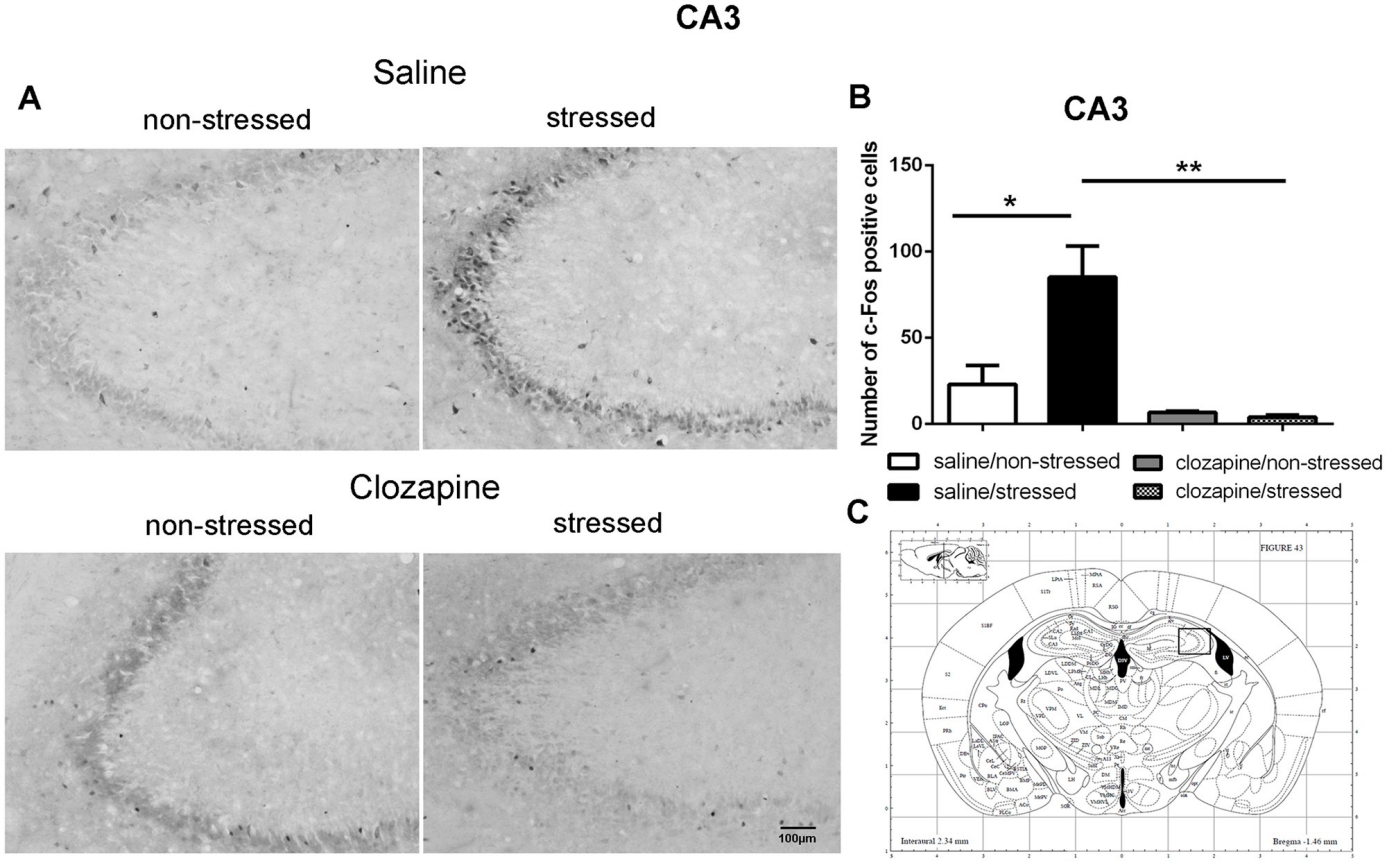
c-Fos immunoreactive cells photomicrographs and quantification in the hippocampus CA3 area. (A) c-Fos staining (dark spots) in coronal sections of CA3 of the four groups: saline/non-stressed, saline/stressed, clozapine/non-stressed, clozapine/stressed. Scale bar: 100 μm. (B) The c-Fos cell counting in the CA3 revealed a higher number of c-Fos cells in saline/stressedgroup (two-way ANOVA, *P <0.05; **P <0.005), data showed as mean ± S.E.M. (C) Representative rat brain atlas drawing [22]. Localization of the brain area selected for the counting of c-Fos cells is indicated in the square.

**Fig 9:**
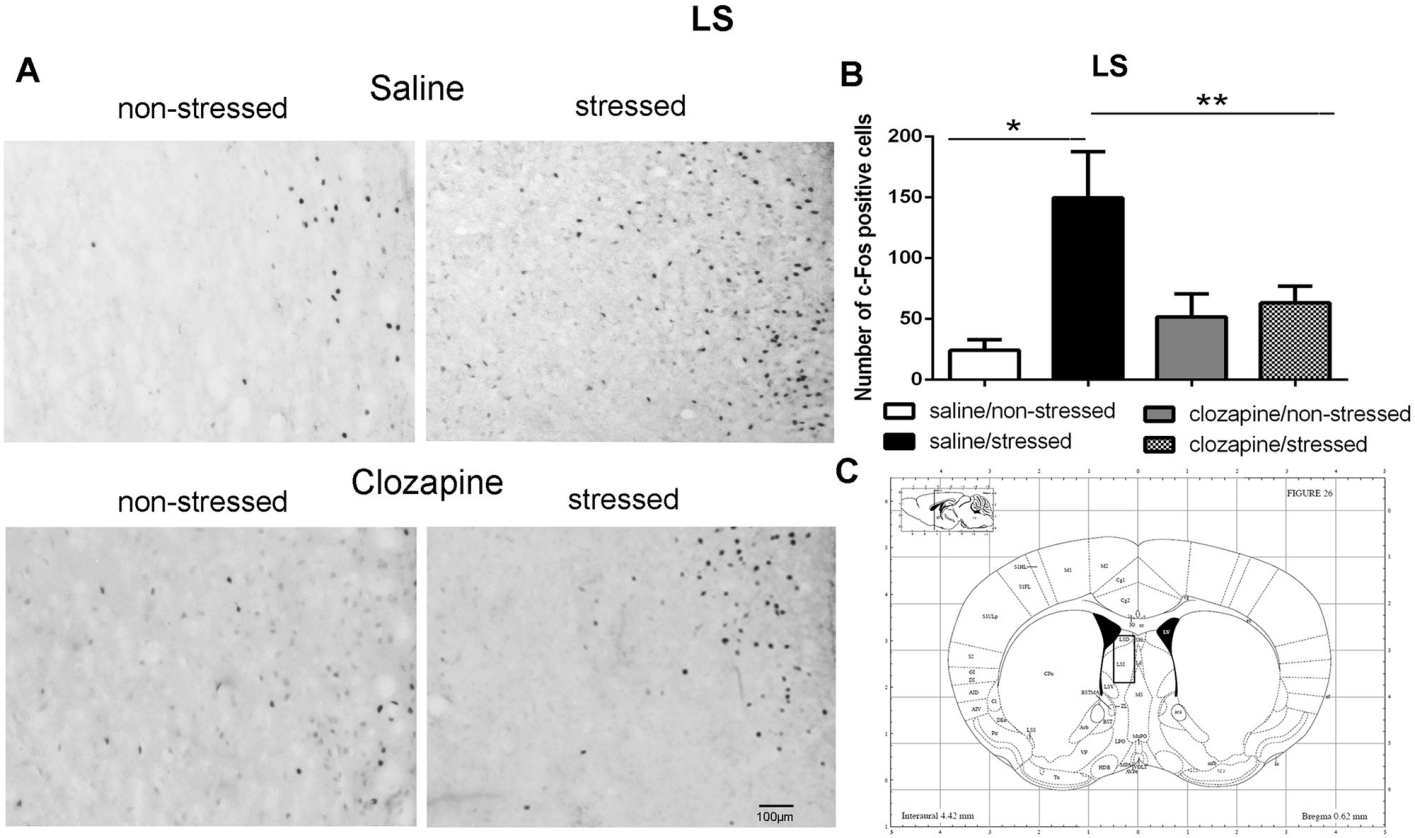
c-Fos immunoreactive cells photomicrographs and quantification in the lateral septum (LS). (A) c-Fos staining (dark spots) in coronal sections of LS of the four groups: saline/non-stressed, saline/stressed, clozapine/non-stressed, clozapine/stressed. Scale bar: 100 μm. (B) The c-Fos cell counting in the LS revealed a higher number of c-Fos cells in saline/stressed group (two-way ANOVA, *P <0.05; **P <0.005), data showed as mean ± S.E.M. (C) Representative rat brain atlas drawing [22]. Localization of the brain area selected for the counting of c-Fos cells is indicated in the square.

**Fig 10:**
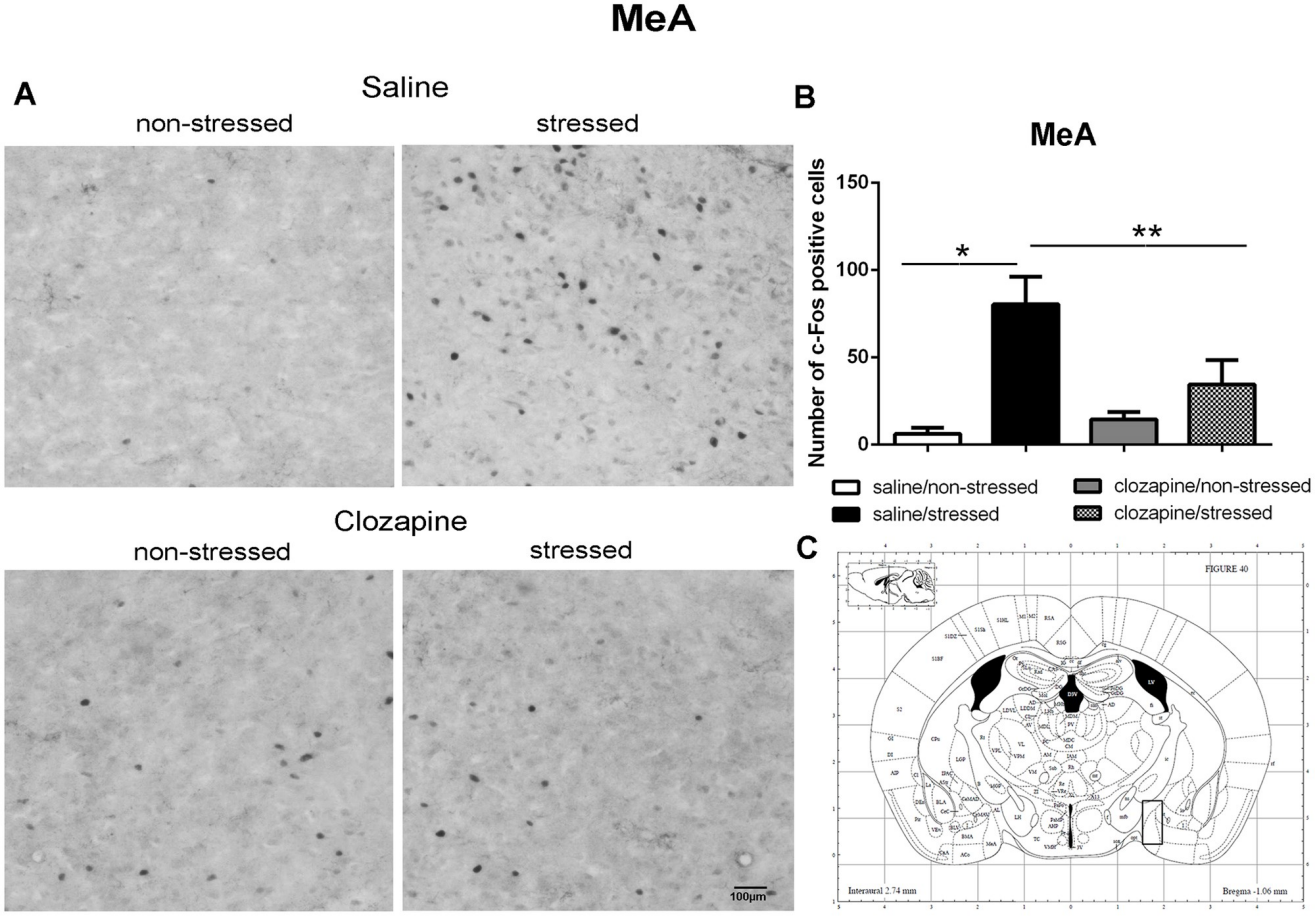
c-Fos immunoreactive cells photomicrographs and quantification in the medial amygdala (MeA). (A) c-Fos staining (dark spots) in coronal sections of MeA of the four groups: saline/non-stressed, saline/stressed, clozapine/non-stressed, clozapine/stressed. Scale bar: 100 μm. (B) The c-Fos cell counting in the MeA revealed a higher number of c-Fos cells in saline/stressed group (two-way ANOVA, *P <0.05; **P <0.005), data showed as mean ± S.E.M. (C) Representative rat brain atlas drawing [22]. Localization of the brain area selected for the counting of c-Fos cells is indicated in the square.

Two-way ANOVA revealed a significant main effect of the condition factor on the following areas: PVN [F (1,16) = 19.90; P < 0.001; 3A,B,C], MnR [F (1,16) = 8.49; P = 0.017; 4A,B,C], mPFC [F (1,16) = 12.15; P < 0.001; 5A,B,C], cPFC [F (1,16) = 10.85; P < 0.001; 6A,B,C], NaC [F (1,16) = 12.68; P < 0.001; 7A,B,C], CA3 [F (1,16) = 7.65; P = 0.000; 8A,B,C], LS [F (1,16) = 7.24; P = 0.029; 9A,B,C] and MeA [F (1,16) = 14.93; P < 0.001; 10A,B,C]. *Post hoc* test showed that the saline/stressed group presented an increase in c-Fos-ir in comparison to the saline/non-stressed group, demonstrating a significant effect of acute restraint stress. Regarding the treatment factor, a significant effect was observed in the same brain regions: PVN [F (1,16) = 0.19; P = 0.038; 3A,B,C], MnR [F (1,16) = 3.53; P = 0.023;4A,B,C], mPFC [F (1,16) = 6.07; P = 0.006; 5A,B,C], cPFC [F (1,16) = 1.90; P = 0.002;6A,B,C], NaC [F (1,16) = 0.14; P = 0.016; 7A,B,C], CA3 [F (1,16) = 20.57; P = 0.000; 8A,B,C], LS [F (1,16) = 1.34; < 0.001; 9A,B,C] and MeA [F (1,16) = 2.35; P = 0.017; 10A,B,C]. *Post hoc* test indicated that the clozapine/stressed group presented a decrease in c-Fos-ir in comparison to the saline/stressed group, demonstrating that pre-treatment with clozapine prevented the effect of acute restraint stress. It was observed a significant interaction between the two factors indicating that the pretreatment with clozapine was effective in reversing the effects of stress on c-Fos expression in the brain areas analyzed: PVN [F (1,16) = 7.60; P = 0.014; 3A,B,C], MnR [F (1,16) = 5.52; P = 0.032; 4A,B,C], mPFC [F (1,16) = 9.66; P = 0.007; 5A,B,C], cPFC [F (1,16) = 6.76; P = 0.019; 6A,B,C], NaC [F (1,16) = 17.48; P < 0.001; 7A,B,C], CA3 [F (1,16) = 9.16; P = 0.008; 8A,B,C], LS [F (1,16) = 5.03; P = 0.039; 9A,B,C] and MeA [F (1,16) = 4.94; P = 0.040;10A,B,C].

For the negative tissue control of c-Fos staining, a naïve control animal (not manipulated and receiving no injection) was used in order to verify any possible non-specific binding of the antibody or false positive results, and no neurons labeled for c-Fos were found.

## 4. Discussion

The behavioral effects of acute restraint stress on the SI test showed that the saline/stressed group presented social withdrawal with a significant reduction in contacts duration. Saline/stressed rats spent less time in social interaction and showed a decrease in the locomotor activity in comparison to the saline/non-stressed group. Previous study carried out in our laboratory showed that 15 min of acute restraint stress increased serum corticosterone level in the stressed rats compared to the non-stressed (P = 0.016), demonstrating that the stress protocol used here effectively activated the HPA axis, an important physiological component of the stress response [9].

Pretreatment with clozapine, prior to restraint, prevented social withdrawal facilitating social behavior in the clozapine/stressed group in comparison to the saline/stressed group. There was no significant difference among the saline/non-stressed and clozapine/non-stressed groups, in the total duration of contacts or in locomotor activity. This is a relevant result since show that treatment with clozapine *per se* did not affect normal social interaction behavior in both non-stressed conditions and discards any motor effect of clozapine treatment.

There are some studies concerning the effect of atypical antipsychotics under stress conditions. For instance, Becker and Grecksch [24] showed that a single injection of clozapine induced social interaction deficit in sham-lesioned rats compared to control rats, in the ventral hippocampus lesion model, while subchronic administration of clozapine did not affect social behavior. Other authors showed that clozapine failed to reverse social interaction deficit induced by acute [25] or 3 days [13] phencyclidine (PCP) treatment in male Wistar rats. Sams-Dodd et al. [26], demonstrated that clozapine did not ameliorate social interaction deficits in the neonatal hippocampal lesion rat model.

In the present study, the treatment with the antipsychotic clozapine in rats submitted to a single episode of stress, attenuated deficits in social interaction and prevented the activation of stress-related brain areas. Since clozapine is used clinically as an antipsychotic, the results of the present study may help to clarify the mechanism by which clozapine is effective in patients with schizophrenia.

Corroborating the behavioral data, the immunohistochemical investigation performed herein showed increased Fos-ir expression in the PVN, MnR, mPFC, cPFC, HPC, LS, NaC and MeA in all saline/stressed groups. Conversely, pre-treatment with clozapine, prevented the increase of Fos-ir expression in these structures. Based on these findings we can suppose that clozapine exerted a preventive effect in the social interaction deficit, at least in part, by blocking the effect of restraint stress, demonstrating that clozapine may influence the dynamic response of the brain to an acute challenge. However, to confirm this hypothesis, an additional experiment should be carried out during which behavior and neurochemistry will be investigated in the same group of rats, in order to establish a significant correlation between changes in c-Fos immunoreactivity and social interaction.

Several studies suggest that clozapine, *per se*, alters c-Fos expression in several brain regions, not only increasing [27–31] but also decreasing expression [32, 33] or even without showing any effect on it [34]. Although clozapine appears to produce a long-term effect on neural metabolism, the mechanisms by which it selectively activates c-Fos in brain areas are not yet fully understood [30, 31]. It has been proposed that clozapine can induce c-Fos expression in the forebrain regions related to the action of antipsychotics such as NaC, LS and mPFC, but not in the dorsal striatum [27, 29, 34], reducing positive and negative symptoms of schizophrenia with low extra-pyramidal side effect [28, 29, 35]. Clozapine also induces c-Fos expression in brain regions involved in stress regulation such as the PVN [34]. In the present study, clozapine increased c-Fos expression in the PVN and the LS, reduced it in the HPC and showed no effect in the MnR, mPFC, cPFC, NaC and MeA when we compared the groups saline and clozapine in the non-stressed condition (data not shown). The data suggest that clozapine has a different impact on the neuronal activity of these forebrain regions and modified their stress response to acute restraint. Several factors may have influenced our results, including the type of stimulus used, the temporal window between clozapine administration and the applied stimulus, as well as other aspects such as the fact that clozapine was injected before giving the stress stimulus, may have altered the basal levels of c-Fos-ir. Interestingly, although clozapine, *per se*, can increase neuronal activation, as detected by c-Fos, this increase was not as robust as the one observed in the saline/stressed group. Based on the idea that the expression of c-Fos helped identifying neurons that mediate stress responses in the various brain regions, it is interesting to note that clozapine was able to prevent an increase of c-Fos-ir even though it was given before the stressor, unlike other study where clozapine treatment was administered concurrently with the stressor [36] reversing c-Fos expression in different brain regions.

In our study, stressed rats presented a reduced number of c-Fos expression in the PVN when treated with clozapine, which raises the possibility that one of the mechanisms of its antipsychotic action is the suppression of the HPA responses. Indeed, the important involvement of the PVN in HPA function, essential for the stress response, has already been well documented for a review see [37, 38]. Acute single episode of stress, such as immobilization, elicit the induction of c-Fos expression mainly in the PVN, activates the HPA axis and increases secretion of ACTH from the anterior pituitary [39]. Moreover, Osacka et al. [34] showed increased c-Fos expression in the PVN in rats exposed to a single forced swimming episode 10 min in duration. Increased c-Fos-ir in the PVN, induced by acute restraint stress, occurs mostly in the region that contains corticotrophin releasing factor (CRF) neurons, the dorsal parvocellular region, and increases plasma ACTH level [39, 40]. It is possible that, at least in part, this activation is mediated by serotonergic neurotransmission since the parvocellular divisions of the PVN receives serotonergic innervation from the MnR, the major serotonergic cell group of the brainstem [41–43]. Indeed, serotonin (5-HT) activates the HPA axis [44] by activating 5-HT_2A_ receptors on PVN neurons [45] stimulating ACTH and corticosterone secretion [37, 43, 46]. Thus, it seems reasonable to assume that reduced MnR and PVN c-Fos-ir induced by clozapine blocking of 5-HT_2A_ receptors in these structures contribute to reduce HPA activity, and thereafter to prevent the deficit of social interaction observed in the clozapine/stressed group. Additionally, these data suggest that clozapine can modulate the brain reactivity to stress exposure.

As mentioned before, in this study, acute restraint stress enhanced c-Fos-ir in the mPFC, cPFC, HPC, LS, and MeA with clozapine blocking the effects of stress in these brain areas as well. Interestingly, 5-HT pathways heavily innervate these limbic forebrain structures [42, 47] suggesting a common site for the antipsychotic action of neuroleptics [48]. Some reports corroborate this proposition: i) the mPFC has an important function in mediating responses physiologically adaptive induced by stress [49]. Studies showed that restraint stress increases c-Fos expression [50] and 5-HT levels [51] in the mPFC which densely express 5-HT_2A_ receptor [52–54]. The acute administration of clozapine significantly reduces the expression of 5-HT2A receptor in the PFC [55]; ii) the mPFC sends projections to the CA3 and MeA [49, 56, 57] that in turns is innervated by the PVN, providing an anatomical substrate for the mPFC actions on the PVN [58]. In addition, 10 min of immobilization in rats elevated extracellular levels of 5-hydroxyindoleacetic acid (5-HIAA) a 5-HT metabolite in the PFC, CA3 and MnR [59]; (iii) LS receives pronounced projections from the MnR [60], and innervates the peri-PVN region [61] participating in the regulation of the HPA axis activity [62]; (iv) NaC also receives serotonergic innervation from the MnR [63] and induction of c-Fos was observed in the NaC and cPFC of rats after 30 min of restraint stress [50]; (v) mPFC, HPC, LS and MeA are implicated in cognitive/affective responses to stress [8, 64, 65] and in endocrine regulations through the modulation of the HPA activity [8, 37, 66–68]. This HPA- corticolimbic circuits appear to be recruited in the stress response to acute restraint. Thus, the effect of acute restraint stress on neuronal activity were examined in brain regions that are known to regulate the HPA-axis, including the cerebral cortex, hippocampus, hypothalamus, septum and amygdala.

Direct correlation between stress and psychosis has been suggested for several reasons: (i) negative symptoms severity are related to ACTH and/or cortisol plasma level in patients with schizophrenia [4, 69]; (ii) acute stress triggers psychotic symptoms in patients with schizophrenia [70, 71]; (iii) atypical antipsychotics decrease HPA activity leading to a reduction in ACTH as well as cortisol secretion in patients with schizophrenia [72]; (iv) withdrawal of atypical antipsychotics of patients with schizophrenia increases cortisol plasma level and it is related to negative symptoms [73]. All these reports, together with the present data, suggest that the protective effect of antipsychotics may be achieved by reducing the biological stress response and implying that the same pathway may be activated both in psychosis and stress [74].

Finally, our results demonstrated that exposure of rats to acute restraint stress induced social withdrawal and c-Fos-ir in the PVN, MnR, mPFC, cPFC, HPC, LS, NaC and MeA. Pre-treatment with clozapine, blocked c-Fos-ir in these limbic forebrain areas preventing social withdrawal. Taken together, these results suggest that at least part of clozapine´s therapeutic effects might involve some kind of regulation of the HPA- corticolimbic circuits recruited during stress response to acute restraint. It is possible that clozapine influences the dynamic response of these brain regions to an acute challenge. Further experiments will be done to confirm this hypothesis.

## Supporting information

S1 File (DOCX)
