# Supplementary material for: Clozapine prevented social interaction deficits and reduced c-Fos immunoreactivity expression in several brain areas of rats exposed to acute restraint stress
Source: PLoS One. 2022 Mar 3;17(3):e0262728. doi: 10.1371/journal.pone.0262728 (PMC8893644; doi:10.1371/journal.pone.0262728)
Supplement: S1 File — (DOCX) [file pone.0262728.s001.docx]

**Data Set Behavioral study**

| social interaction - duration of contacts (s) |  |  |  |  |  |
| --- | --- | --- | --- | --- | --- |
|  |  |  |  |  |  |
| **saline/non-stressed** |  |  |  | **saline/stressed** |  |
|  |  |  |  |  |  |
| 1 | 56,45 |  |  | 1 | 79,67 |
| 2 | 120,51 |  |  | 2 | 22,62 |
| 3 | 137,93 |  |  | 3 | 8 |
| 4 | 84,67 |  |  | 4 | 54,05 |
| 5 | 157,35 |  |  | 5 | 31,62 |
| 6 | 134,72 |  |  | 6 | 46,64 |
| 7 | 91,89 |  |  | 7 | 33,61 |
| 8 | 174,56 |  |  | 8 | 22,81 |
| 9 | 119,31 |  |  | 9 | 40,43 |
| 10 | 127,52 |  |  | 10 | 15,41 |
| **MEAN** | **120,491** |  |  | **MEAN** | **35,486** |
| **SD** | **35,02672** |  |  | **SD** | **20,94678** |
| **SEM** | **11,07642** |  |  | **SEM** | **6,623954** |
|  |  |  |  |  |  |
|  |  |  |  |  |  |
|  |  |  |  |  |  |
| **clozapine/non-stressed** |  |  |  | **clozapine/stressed** |  |
|  |  |  |  |  |  |
| 1 | 198,99 |  |  |  | 60,66 |
| 2 | 225,82 |  |  |  | 51,25 |
| 3 | 185,58 |  |  |  | 163,36 |
| 4 | 69,86 |  |  |  | 64,06 |
| 5 | 110,3 |  |  |  | 127,52 |
| 6 | 85,68 |  |  |  | 243,84 |
| 7 | 92,69 |  |  |  | 68,26 |
| 8 | 176,77 |  |  |  | 55,25 |
| 9 | 128,52 |  |  |  | 95,09 |
| 10 | 189,98 |  |  |  | 125,12 |
| **MEAN** | **146,419** |  |  | **MEAN** | **105,441** |
| **SD** | **55,25982** |  |  | **SD** | **61,50586** |
| **SEM** | **17,47469** |  |  | **SEM** | **19,44986** |

| locomotor activity – number of crossings | |  | | | | |  | | | |  | | | |  | | | | |  | | | | |  |  |
| --- | --- | --- | --- | --- | --- | --- | --- | --- | --- | --- | --- | --- | --- | --- | --- | --- | --- | --- | --- | --- | --- | --- | --- | --- | --- | --- |
|  | |  | | | | |  | | | |  | | | |  | | | | |  | | | | |  |  |
|  | |  | | | |  | | | |  | | |  | | | | | |  | | | | |  |  |  |
| **saline/non-stressed** | |  | | | |  | | | |  | | | **saline/stressed** | | | | | |  | | | | |  |  |  |
|  | |  | | | |  | | | |  | | |  | | | | | |  | | | | |  |  |  |
| 1 | | 81 | | | |  | | | |  | | | 1 | | | | | | 78 | | | | |  |  |  |
| 2 | | 246 | | | |  | | | |  | | | 2 | | | | | | 81 | | | | |  |  |  |
| 3 | | 251 | | | |  | | | |  | | | 3 | | | | | | 52 | | | | |  |  |  |
| 4 | | 193 | | | |  | | | |  | | | 4 | | | | | | 83 | | | | |  |  |  |
| 5 | | 269 | | | |  | | | |  | | | 5 | | | | | | 61 | | | | |  |  |  |
| 6 | | 142 | | | |  | | | |  | | | 6 | | | | | | 62 | | | | |  |  |  |
| 7 | | 198 | | | |  | | | |  | | | 7 | | | | | | 81 | | | | |  |  |  |
| 8 | | 174 | | | |  | | | |  | | | 8 | | | | | | 68 | | | | |  |  |  |
| 9 | | 230 | | | |  | | | |  | | | 9 | | | | | | 71 | | | | |  |  |  |
| 10 | | 202 | | | |  | | | |  | | | 10 | | | | | | 148 | | | | |  |  |  |
| **MEAN** | | **198,6** | | | |  | | | |  | | | **MEAN** | | | | | | **78,5** | | | | |  |  |  |
| **SD** | | **56,38794** | | | |  | | | |  | | | **SD** | | | | | | **26,47955** | | | | |  |  |  |
| **SEM** | | **17,83143** | | | |  | | | |  | | | **SEM** | | | | | | **46,80171** | | | | |  |  |  |
|  | |  | | | |  | | | |  | | |  | | | | | |  | | | | |  |  |  |
|  | |  | | | |  | | | |  | | |  | | | | | |  | | | | |  |  |  |
|  | |  | | | |  | | | |  | | |  | | | | | |  | | | | |  |  |  |
| **clozapine/non-stressed** | |  | | | |  | | | |  | | | **clozapine/stressed** | | | | | |  | | | | |  |  |  |
|  | |  | | | |  | | | |  | | |  | | | | | |  | | | | |  |  |  |
| 1 | | 199 | | | |  | | | |  | | | 1 | | | | | | 198 | | | | |  |  |  |
| 2 | | 225 | | | |  | | | |  | | | 2 | | | | | | 156 | | | | |  |  |  |
| 3 | | 185 | | | |  | | | |  | | | 3 | | | | | | 152 | | | | |  |  |  |
| 4 | | 139 | | | |  | | | |  | | | 4 | | | | | | 151 | | | | |  |  |  |
| 5 | | 110 | | | |  | | | |  | | | 5 | | | | | | 172 | | | | |  |  |  |
| 6 | | 126 | | | |  | | | |  | | | 6 | | | | | | 129 | | | | |  |  |  |
| 7 | | 159 | | | |  | | | |  | | | 7 | | | | | | 182 | | | | |  |  |  |
| 8 | | 176 | | | |  | | | |  | | | 8 | | | | | | 194 | | | | |  |  |  |
| 9 | | 128 | | | |  | | | |  | | | 9 | | | | | | 165 | | | | |  |  |  |
| 10 | | 194 | | | |  | | | |  | | | 10 | | | | | | 168 | | | | |  |  |  |
| **MEAN** | | **164,1** | | | |  | | | |  | | | **MEAN** | | | | | | **166,7** | | | | |  |  |  |
| **SD** | | **37,61929** | | | |  | | | |  | | | **SD** | | | | | | **21,05575** | | | | |  |  |  |
| **SEM** | | **11,89626** | | | |  | | | |  | | | **SEM** | | | | | | **6,658412** | | | | |  |  |  |
| **Immunohistochemical study** | |  | | | |  | | | |  | | |  | | | | | |  | | | | |  |  |  |
| c-Fos cell counting | | |  | | | | | |  | | | | | | |  | | | | |  | | | | |  |
|  | | | | |  | | | | | | |  | | | | | |  | | | | |  | | | |
|  | | | | |  | | | | | | |  | | | | | |  | | | | |  | | | |
| CONDITION | | | | | RAT | | | | | | | PVN | | | | | | mPFC | | | | | cPFC | | | |
| SALINE/NON-STRESSED | | | | | 6 | | | | | | | 7 | | | | | | 3 | | | | | 23 | | | |
| SALINE/NON-STRESSED | | | | | 15 | | | | | | | 5 | | | | | | 19 | | | | | 65 | | | |
| SALINE/NON-STRESSED | | | | | 17 | | | | | | | 19 | | | | | | 7 | | | | | 160 | | | |
| SALINE/NON-STRESSED | | | | | 20 | | | | | | | 20 | | | | | | 7 | | | | | 72 | | | |
| SALINE/NON-STRESSED | | | | | 24 | | | | | | | 11 | | | | | | 9 | | | | | 7 | | | |
| **MEAN** | | | | |  | | | | | | | **12,40** | | | | | | **9,00** | | | | | **65,40** | | | |
| **SD** | | | | |  | | | | | | | **6,12** | | | | | | **5,37** | | | | | **53,29** | | | |
| **SEM** | | | | |  | | | | | | | **2,74** | | | | | | **2,40** | | | | | **23,83** | | | |
|  | | | | |  | | | | | | |  | | | | | |  | | | | |  | | | |
|  | | | | |  | | | | | | |  | | | | | |  | | | | |  | | | |
| CONDITION | | | | | RAT | | | | | | | PVN | | | | | | mPFC | | | | | cPFC | | | |
| SALINE/STRESSED | | | | | 5 | | | | | | | 139 | | | | | | 56 | | | | | 290 | | | |
| SALINE/STRESSED | | | | | 11 | | | | | | | 151 | | | | | | 50 | | | | | 753 | | | |
| SALINE/STRESSED | | | | | 12 | | | | | | | 240 | | | | | | 68 | | | | | 1084 | | | |
| SALINE/STRESSED | | | | | 21 | | | | | | | 326 | | | | | | 49 | | | | | 846 | | | |
| SALINE/STRESSED | | | | | 23 | | | | | | | 300 | | | | | | 96 | | | | | 807 | | | |
| **MEAN** | | | | |  | | | | | | | **231,20** | | | | | | **63,80** | | | | | **756,00** | | | |
| **SD** | | | | |  | | | | | | | **75,80** | | | | | | **17,46** | | | | | **259,02** | | | |
| **SEM** | | | | |  | | | | | | | **33,90** | | | | | | **7,81** | | | | | **115,84** | | | |
|  | | | | |  | | | | | | |  | | | | | |  | | | | |  | | | |
|  | | | | |  | | | | | | |  | | | | | |  | | | | |  | | | |
| CONDITION | | | | | RAT | | | | | | | PVN | | | | | | mPFC | | | | | cPFC | | | |
| CLOZAPINE/NON-STRESSED | | | | | 8 | | | | | | | 261 | | | | | | 55 | | | | | 69 | | | |
| CLOZAPINE/NON-STRESSED | | | | | 14 | | | | | | | 26 | | | | | | 25 | | | | | 84 | | | |
| CLOZAPINE/NON-STRESSED | | | | | 18 | | | | | | | 24 | | | | | | 22 | | | | | 57 | | | |
| CLOZAPINE/NON-STRESSED | | | | | 22 | | | | | | | 14 | | | | | | 1 | | | | | 20 | | | |
| CLOZAPINE/NON-STRESSED | | | | | 26 | | | | | | | 8 | | | | | | 16 | | | | | 25 | | | |
| **MEAN** | | | | |  | | | | | | | **66,60** | | | | | | **23,80** | | | | | **51,00** | | | |
| **SD** | | | | |  | | | | | | | **97,42** | | | | | | **17,66** | | | | | **24,84** | | | |
| **SEM** | | | | |  | | | | | | | **43,57** | | | | | | **7,90** | | | | | **11,11** | | | |
|  | | | | |  | | | | | | |  | | | | | |  | | | | |  | | | |
|  | | | | |  | | | | | | |  | | | | | |  | | | | |  | | | |
| CONDITION | | | | | RAT | | | | | | | PVN | | | | | | mPFC | | | | | cPFC | | | |
| CLOZAPINE/STRESSED | | | | | 7 | | | | | | | 194 | | | | | | 50 | | | | | 171 | | | |
| CLOZAPINE/STRESSED | | | | | 13 | | | | | | | 137 | | | | | | 56 | | | | | 110 | | | |
| CLOZAPINE/STRESSED | | | | | 16 | | | | | | | 18 | | | | | | 3 | | | | | 280 | | | |
| CLOZAPINE/STRESSED | | | | | 19 | | | | | | | 66 | | | | | | 4 | | | | | 35 | | | |
| CLOZAPINE/STRESSED | | | | | 25 | | | | | | | 20 | | | | | | 14 | | | | | 7 | | | |
| **MEAN** | | | | |  | | | | | | | **87,00** | | | | | | **25,40** | | | | | **120,60** | | | |
| **SD** | | | | |  | | | | | | | **68,76** | | | | | | **22,94** | | | | | **98,28** | | | |
| **SEM** | | | | |  | | | | | | | **30,75060975** | | | | | | **10,2590448** | | | | | **43,9514277** | | | |
|  | | | | |  | | | | | | |  | | | | | |  | | | | |  | | | |
|  | |  | | | | |  | | | |  | | | |  | | | | |  | | | | |  |  |
|  | |  | | | | |  | | | |  | | | |  | | | | |  | | | | |  |  |
| CONDITON | NaC | | | MnR | | | | CA3 | | | | | | LS | | | MeA | | | | |  |  |  |  |  |
| SAL/NON-STRESSED | 6 | | | 1 | | | | 10 | | | | | | 19 | | | 2 | | | | |  |  |  |  |  |
| SAL/NON-STRESSED | 9 | | | 1 | | | | 4 | | | | | | 2 | | | 2 | | | | |  |  |  |  |  |
| SAL/NON-STRESSED | 4 | | | 2 | | | | 65 | | | | | | 56 | | | 3 | | | | |  |  |  |  |  |
| SAL/NON-STRESSED | 10 | | | 6 | | | | 31 | | | | | | 37 | | | 22 | | | | |  |  |  |  |  |
| SAL/NON-STRESSED | 13 | | | 3 | | | | 5 | | | | | | 7 | | | 2 | | | | |  |  |  |  |  |
| **MEAN** | **8,40** | | | **2,60** | | | | **23,00** | | | | | | **24,20** | | | **6,20** | | | | |  |  |  |  |  |
| **SD** | **3,14** | | | **1,85** | | | | **25,89** | | | | | | **19,95** | | | **7,91** | | | | |  |  |  |  |  |
| **SEM** | **1,40** | | | **0,83** | | | | **11,58** | | | | | | **8,92** | | | **3,54** | | | | |  |  |  |  |  |
|  |  | | |  | | | |  | | | | | |  | | |  | | | | |  |  |  |  |  |
| CONDITION | NaC | | | MnR | | | | CA3 | | | | | | LS | | | MeA | | | | |  |  |  |  |  |
| SAL/STRESSED | 29 | | | 8 | | | | 35 | | | | | | 125 | | | 50 | | | | |  |  |  |  |  |
| SAL/STRESSED | 190 | | | 19 | | | | 73 | | | | | | 58 | | | 97 | | | | |  |  |  |  |  |
| SAL/STRESSED | 48 | | | 17 | | | | 146 | | | | | | 311 | | | 112 | | | | |  |  |  |  |  |
| SAL/STRESSED | 63 | | | 13 | | | | 78 | | | | | | 133 | | | 27 | | | | |  |  |  |  |  |
| SAL/STRESSED | 54 | | | 12 | | | | 94 | | | | | | 122 | | | 116 | | | | |  |  |  |  |  |
| **MEAN** | **76,80** | | | **13,80** | | | | **85,20** | | | | | | **149,80** | | | **80,40** | | | | |  |  |  |  |  |
| **SD** | **57,69** | | | **3,87** | | | | **40,29** | | | | | | **84,95** | | | **35,54** | | | | |  |  |  |  |  |
| **SEM** | **25,80** | | | **1,73** | | | | **18,02** | | | | | | **37,99** | | | **15,90** | | | | |  |  |  |  |  |
|  |  | | |  | | | |  | | | | | |  | | |  | | | | |  |  |  |  |  |
| CONDITION | NaC | | | MnR | | | | CA3 | | | | | | LS | | | MeA | | | | |  |  |  |  |  |
| CLOZ/NON-STRESSED | 30 | | | 3 | | | | 7 | | | | | | 115 | | | 4 | | | | |  |  |  |  |  |
| CLOZ/NON-STRESSED | 20 | | | 7 | | | | 5 | | | | | | 33 | | | 27 | | | | |  |  |  |  |  |
| CLOZ/NON-STRESSED | 41 | | | 2 | | | | 10 | | | | | | 88 | | | 24 | | | | |  |  |  |  |  |
| CLOZ/NON-STRESSED | 16 | | | 4 | | | | 6 | | | | | | 19 | | | 11 | | | | |  |  |  |  |  |
| CLOZ/NON-STRESSED | 32 | | | 2 | | | | 6 | | | | | | 4 | | | 7 | | | | |  |  |  |  |  |
| **MEAN** | **27,80** | | | **3,60** | | | | **6,80** | | | | | | **51,80** | | | **14,60** | | | | |  |  |  |  |  |
| **SD** | **8,91** | | | **1,85** | | | | **1,92** | | | | | | **42,47** | | | **9,22** | | | | |  |  |  |  |  |
| **SEM** | **3,98** | | | **0,83** | | | | **0,86** | | | | | | **18,99** | | | **4,12** | | | | |  |  |  |  |  |
|  |  | | |  | | | |  | | | | | |  | | |  | | | | |  |  |  |  |  |
| CONDITION | NaC | | | MnR | | | | CA3 | | | | | | LS | | | MeA | | | | |  |  |  |  |  |
| CLZ/STRESSED | 34 | | | 19 | | | | 2 | | | | | | 121 | | | 94 | | | | |  |  |  |  |  |
| CLZ/STRESSED | 32 | | | 1 | | | | 6 | | | | | | 55 | | | 28 | | | | |  |  |  |  |  |
| CLZ/STRESSED | 7 | | | 2 | | | | 2 | | | | | | 64 | | | 31 | | | | |  |  |  |  |  |
| CLZ/STRESSED | 15 | | | 1 | | | | 2 | | | | | | 41 | | | 6 | | | | |  |  |  |  |  |
| CLZ/STRESSED | 48 | | | 1 | | | | 8 | | | | | | 35 | | | 14 | | | | |  |  |  |  |  |
| **MEAN** | **27,20** | | | **4,80** | | | | **4,00** | | | | | | **63,20** | | | **34,60** | | | | |  |  |  |  |  |
| **SD** | **14,55** | | | **7,11** | | | | **2,82** | | | | | | **30,65** | | | **31,07** | | | | |  |  |  |  |  |
| **SEM** | **6,50784142** | | | **3,1799371** | | | | **1,261142339** | | | | | | **13,706641** | | | **13,895611** | | | | |  |  |  |  |  |
|  | |  | | | | |  | | | |  | | | |  | | | | |  | | | | |  |  |
|  | |  | | | | |  | | | |  | | | |  | | | | |  | | | | |  |  |
|  | |  | | | | |  | | | |  | | | |  | | | | |  | | | | |  |  |
|  | |  | | | | |  | | | |  | | | |  | | | | |  | | | | |  |  |
|  | |  | | | | |  | | | |  | | | |  | | | | |  | | | | |  |  |
|  | |  | | | | |  | | | |  | | | |  | | | | |  | | | | |  |  |
|  | |  | | | | |  | | | |  | | | |  | | | | |  | | | | |  |  |
|  | |  | | | | |  | | | |  | | | |  | | | | |  | | | | |  |  |
|  | |  | | | | |  | | | |  | | | |  | | | | |  | | | | |  |  |
|  | |  | | | | |  | | | |  | | | |  | | | | |  | | | | |  |  |
|  | |  | | | | |  | | | |  | | | |  | | | | |  | | | | |  |  |
|  | |  | | | | |  | | | |  | | | |  | | | | |  | | | | |  |  |
|  | |  | | | | |  | | | |  | | | |  | | | | |  | | | | |  |  |
|  | |  | | | | |  | | | |  | | | |  | | | | |  | | | | |  |  |
|  | |  | | | | |  | | | |  | | | |  | | | | |  | | | | |  |  |
|  | |  | | | | |  | | | |  | | | |  | | | | |  | | | | |  |  |
